# Clinical applications of diffusion weighted imaging in neuroradiology

**DOI:** 10.1007/s13244-018-0624-3

**Published:** 2018-05-30

**Authors:** Marta Drake-Pérez, Jose Boto, Aikaterini Fitsiori, Karl Lovblad, Maria Isabel Vargas

**Affiliations:** 1 Division of Diagnostic and Interventional Neuroradiology of Geneva University Hospitals, DISIM and Faculty of Medicine of Geneva, Rue Gabrielle-Perret-Gentil 4, 1211 Genève 14, Switzerland; 2 Department of Radiology, University Hospital Marqués de Valdecilla – IDIVAL, Santander, Spain

**Keywords:** MRI, DWI, Stroke, Infection, Inflammation

## Abstract

**Abstract:**

Diffusion-weighted imaging (DWI) has revolutionised stroke imaging since its introduction in the mid-1980s, and it has also become a pillar of current neuroimaging. Diffusion abnormalities represent alterations in the random movement of water molecules in tissues, revealing their microarchitecture, and occur in many neurological conditions. DWI provides useful information, increasing the sensitivity of MRI as a diagnostic tool, narrowing the differential diagnosis, providing prognostic information, aiding in treatment planning and evaluating response to treatment. Recently, there have been several technical improvements in DWI, leading to reduced acquisition time and artefacts and enabling the development of diffusion tensor imaging (DTI) as a tool for assessing white matter. We aim to review the main clinical uses of DWI, focusing on the physiological mechanisms that lead to diffusion abnormalities. Common pitfalls will also be addressed.

**Teaching Points:**

• *DWI includes EPI, TSE, RESOLVE or EPI combined with reduced volume excitation.*

• *DWI is the most sensitive sequence in stroke diagnosis and provides information about prognosis.*

• *DWI helps in the detection of intramural haematomas (arterial dissection).*

• *In diffusion imaging, ADC is inversely proportional to tumour cellularity.*

• *DWI and DTI derived parameters can be used as biomarkers in different pathologies.*

## Introduction

Diffusion-weighted imaging (DWI) has revolutionised stroke imaging since its introduction in the mid-1980s, but it has also become a pillar of current neuroimaging. Diffusion abnormalities represent alterations in the random movement of water molecules in tissues, revealing their microarchitecture, and occur in many neurological conditions. DWI provides useful information, increasing the sensitivity of MRI as a diagnostic tool, narrowing the differential diagnosis, providing prognostic information, aiding the treatment planning and evaluating response to treatment.

There have been several technical improvements in DWI, leading to reduced acquisition time and artefacts and enabling the development of diffusion tensor imaging (DTI) as a tool for assessing white matter. DTI allows tractography for surgical planning in brain tumours and other advanced techniques such as intravoxel incoherent motion (IVIM) [1] and kurtosis, which provide information about the complexity of tissues and their different compartments (intravascular, intracellular and extracellular).

We aim to review the main clinical uses of DWI, focusing on the physiological mechanisms that lead to diffusion abnormalities. Common pitfalls will also be addressed.

## DWI sequences used in neuroradiology

Echo-planar imaging (EPI) is the method of choice for clinical diffusion-weighted imaging with MRI because of its low sensitivity to the motion-induced phase errors that occur during diffusion sensitisation of the MR signal. EPI is able to capture an entire image in a very short time. However, this method is prone to artefacts because of susceptibility changes at tissue interfaces and has limited spatial resolution. Major technical advances in diffusion MRI have enabled superior data fidelity, reduced image acquisition time and an improved signal-to-noise ratio (SNR) [2].

Turbo-spin echo (TSE) is an adequate sequence to minimise susceptibility artefacts and distortion, and it increases the signal-to-noise ratio. Unfortunately, it also increases acquisition times and is therefore more sensitive to motion throughout the procedure.

Readout segmentation EPI techniques (e.g. RESOLVE) allow DWI and DTI to be simultaneously generated, thus reducing the level of susceptibility-based distortion compared with the single-shot EPI technique [3]. These also improve the overall quality and spatial resolution of the images, especially when air-tissue interfaces are involved. However, they have longer scanning times and are more sensitive to motion. Therefore, its main clinical use is to study small regions adjacent to air-tissue interfaces such as the inner ear or spinal cord [4].

EPI combined with reduced volume excitation (ZOOMit; Siemens Healthcare, Erlangen, Germany) is a focused excitation method that utilises two orthogonal pulses for slice selection in such a way that only the spins belonging to the intersection of the slab give rise to signal. This obviates the need for oversampling or distortion reduction [4] and allows zoomed fields of view. Advantages of this method are improved image quality, increased image sharpness and reduced distortion, with no additional acquisition time. A major disadvantage is the reduced field of view. As a result, this technique is also useful for the evaluation of small structures, especially if they are close to air-tissue interfaces (orbit or spinal cord).

Liney et al. compared EPI, RESOLVE and EPI combined with reduced volume excitation, concluding that EPI provided significantly higher ADC values than the other two sequences and that RESOLVE had the best repeatability ratio [5].

## DWI, DTI and their derived parameters

Parameters derived from DWI and DTI studies allow a quantitative assessment, which in turn leads to improved accuracy and reproducibility. However, it should be noted that some of these parameters are still subject to clinical validation [6].

ADC is the most widely used parameter derived from the conventional DWI sequence, representing the level of restriction to the motion of water molecules in the extracellular compartment. The term “apparent” relates to the fact that this motion is also influenced by other physiological processes, such as heartbeat, breathing or CSF pulsation. The main advantage of ADC relies on its widespread availability [7].

DTI is based on the application of diffusion gradients in at least six different directions in space, enabling the evaluation of the movement of water molecules in 3D and whether there is a dominant direction to diffusion restriction. This allows tractography studies to be performed, which are multiplanar reconstructions of white matter tracts according to the dominant direction of water movement in each voxel.

DTI provides several quantitative parameters, most of which are useful in the assessment of white matter conditions. Fractional anisotropy (FA) reflects how dominant one particular water movement direction in a voxel is. It varies from 0 to 1 and can be considered a biomarker of axonal integrity (usually decreased in white matter pathologies). Mean diffusivity (MD) represents a more exact value than ADC because it considers the three main directions of water movement. Axial diffusivity (AD) quantifies water movement along the main longitudinal direction, evaluating axonal integrity, while radial diffusivity (RD) reflects myelin integrity (myelin injury leads to increased RD).

As we will discuss, all these quantitative parameters improve the diagnostic possibilities of MR in the evaluation of tumours, demyelinating and infectious diseases, and vascular pathologies.

## Cerebrovascular disease

### Stroke

Brain ischaemia leads to variations in the intra- and extracellular space due to a failure of the Na^+^/K^+^ ATPase and other ionic pumps. Water movement inside the cell becomes more limited resulting in cytotoxic oedema. In addition, the intracellular environment becomes more viscous, further restricting diffusion and decreasing the apparent diffusion coefficient (ADC).

Subsequently, inflammatory mediation factors are released, affecting the integrity of the blood-brain barrier and leading to water extravasation and vasogenic oedema with expansion of the interstitial space and an increase of the total water content of the tissue. Subsequently, ADC values gradually increase to their baseline. In chronic ischaemia gliosis eventually develops, which does not result in restricted diffusion.

**Fig. 1:**
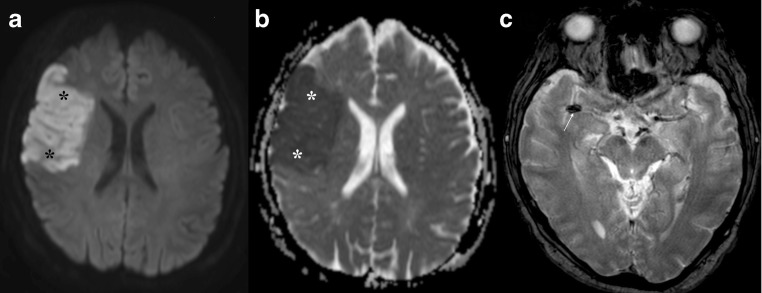
Acute stroke. **a** Increased signal intensity on DWI in the territory of the right middle cerebral artery (MCA), which corresponds to an area of low ADC value in **b** (asterisks), representing the extension of an acute ischaemic stroke. **c** A clot as the aetiology, presenting as a linear hypointense abnormality in the MCA on T2*WI (arrow)

DWI improves detection of ischaemia because it reveals alterations in water diffusion before the net increase of water content in the subacute phase of the infarct. DWI hyperintensity [8, 9] and changes in ADC values [10] can be found within minutes from the onset of ischaemia (Fig. 1). EPI has high sensitivity and specificity in stroke diagnosis, but a negative result does not exclude a vascular aetiology [11]. Stroke in the posterior circulation is more frequently DWI negative than in the anterior circulation (34.9% versus 15.3%, respectively; *p* = 0.0019) [12].

DWI is far more sensitive than conventional imaging in the detection of early ischaemia. T2WI—including fluid-attenuated inversion recovery (FLAIR) imaging—can only detect ischaemia typically 1–4 h later, since signal abnormalities on these sequences relate to the subsequent increase in total water content in the infarcted area [13].

In the setting of acute ischaemia, the mismatch between DWI and FLAIR has been suggested to enable identification of patients with acute ischaemic stroke who are likely to be within 4.5 h of symptom onset with high specificity and high positive predictive value [14], which is useful for identifying candidates for fibrinolysis.

ADC alone can be considered a biomarker of brain ischaemia as it allows differentiation between the two main causes of increased DWI signal in acute stroke [15]: “true” diffusion restriction and T2 signal elongation. ADC values also yield timing information [16]: they start to decrease from the 1st hour, reaching the minimal value around the 24th hour. Subsequently, ADC values increase after the 3rd day, reaching higher values than the surrounding parenchyma after the 10th day. When combined with T2WI signal changes, they allow radiologists to estimate the approximate time of stroke onset. It should however be noted that haemorrhagic transformation may be present, which will affect the signal of the lesion and modify the expected sequence of events.

The area showing diffusion restriction is usually taken as the “infarct core” (final infarct size), but some diffusion abnormalities may be reversible, mainly if there is early (< 4.5 h) spontaneous or therapeutic recanalisation of the occluded vessels [17]. However, the size of the area of diffusion restriction (and the ADC value) correlates with prognosis, and volume measurements and scores (ASPECTS, DRAGON) can be useful tools in predicting the outcome after intravenous thrombolysis [18].

Thrombolytic or endovascular therapies are the treatment options for acute stroke. Selection of patients eligible for these therapies is critical to achieve good outcomes and avoid fatal complications. Both time from stroke symptom onset to treatment and the individual therapeutic time window secondary to variations in collateral circulation determine the long-term functional outcome after stroke. Therefore, imaging—including DWI—has great prognostic value and is crucial to treatment decision making because it allows early diagnosis and an estimation of both the time of onset and the infarct-salvageable brain proportion.

**Fig. 2:**
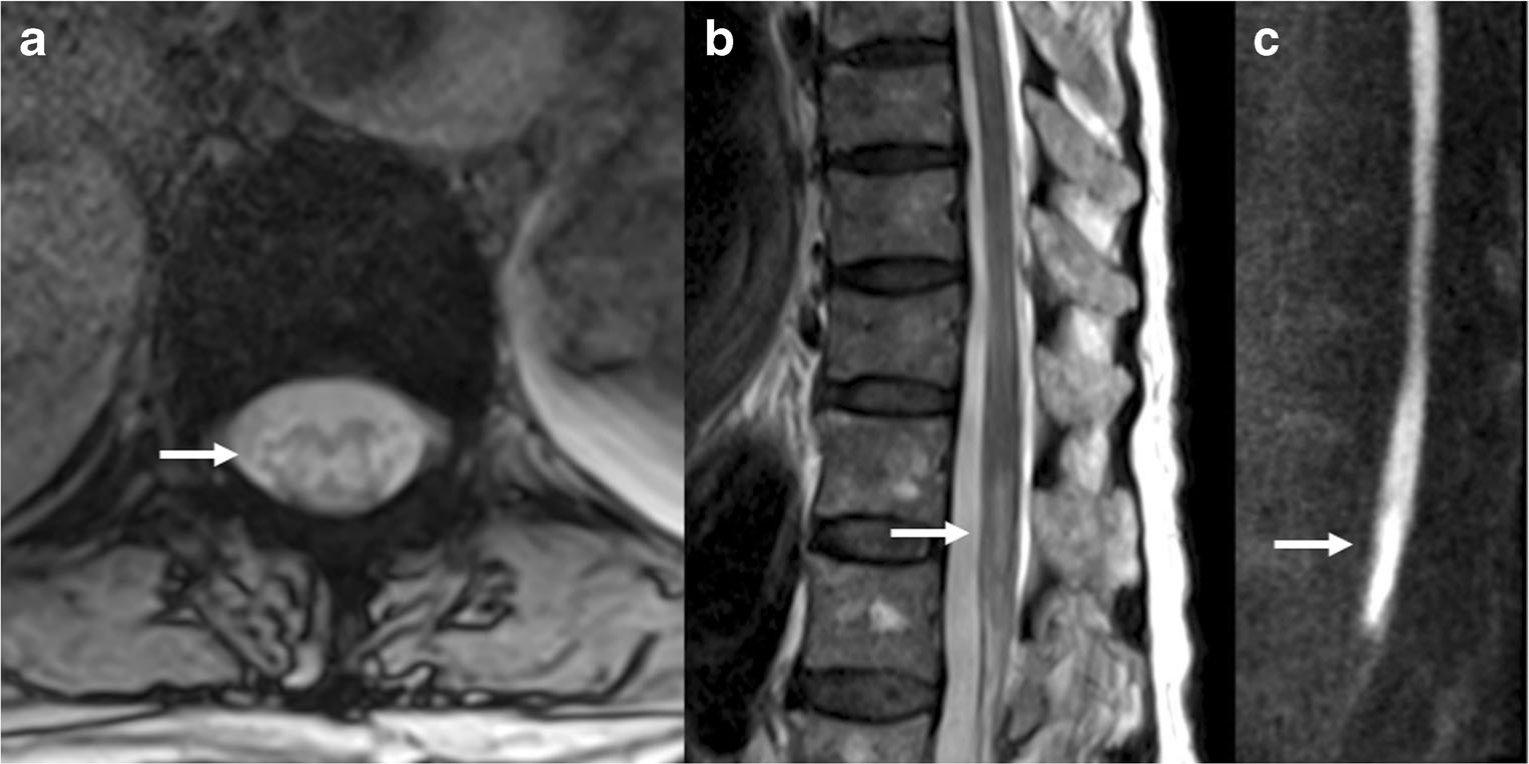
Spinal cord ischaemia. High T2 signal abnormality in the conus medullaris with diffusion restriction on DWI (arrows) compatible with an ischaemic lesion

Despite some technical challenges (need for strong gradients, the size of the spinal cord, flow artefacts), DWI is useful in assessing cord ischaemia. Although signal changes were reported on both T2 and DWI in the majority of cases, DWI showed a clear benefit over conventional MRI in some patients, in whom T2 failed to identify an abnormality [19, 20]. According to Thurnher et al., the earliest changes on DWI in spinal cord ischaemia can be seen 3 h after symptom onset [21] An imaging protocol with DWI of the spinal cord with maximum b values between 500 and 700 s/mm^2^ and a slice thickness of 3 mm is recommended [22] (Fig. 2).

**Table:** 

Key points	Ischaemia leads to DWI changes within minutes of symptom onset
Pearls	DWI helps in: Diagnosis (most sensitive sequence) Prognosis and treatment planning (DWI aids in “dating” the stroke and in achieving better patient selection)
Pitfalls	False negatives for posterior circulation strokes possible

### Transient ischaemic attack (TIA)

The new definition of TIA is based on a biological concept (tissue injury). A TIA is characterised by a transient episode of neurological impairment caused by focal ischaemia without acute infarction [23].

Previously, TIA was defined based on arbitrary timing (duration ≤ 24 h). However, permanent tissue damage (infarction) is possible even when focal transient neurological symptoms last < 1 h [24]. Additionally, approximately 50% of patients with classically defined TIA syndromes have corresponding ischaemic lesions on brain MRI diffusion- or perfusion-weighted imaging. Therefore, whether a symptomatic ischaemic episode will result in ischaemic infarction cannot be based solely on time duration.

The current definition takes imaging into account as DWI must be normal for the diagnosis of TIA to be upheld. If MRI shows areas of restricted diffusion in a patient whose symptoms have resolved and lasted less than 24 h, the term cerebral infarction with transient symptoms is preferred [25]. Once a morphological change has been demonstrated on imaging, the risk of full-blown stroke is higher, and treatment should be more aggressive.

**Table:** 

Key points	New definition that takes imaging into account (DWI -)
Pearls	If DWI +, the term cerebral infarction with transient symptoms is preferred (more aggressive treatment)
Pitfalls	The duration of ischaemic symptoms does not predict whether a symptomatic ischaemic event will result in ischaemic infarction

### Arterial dissection

**Fig. 3:**
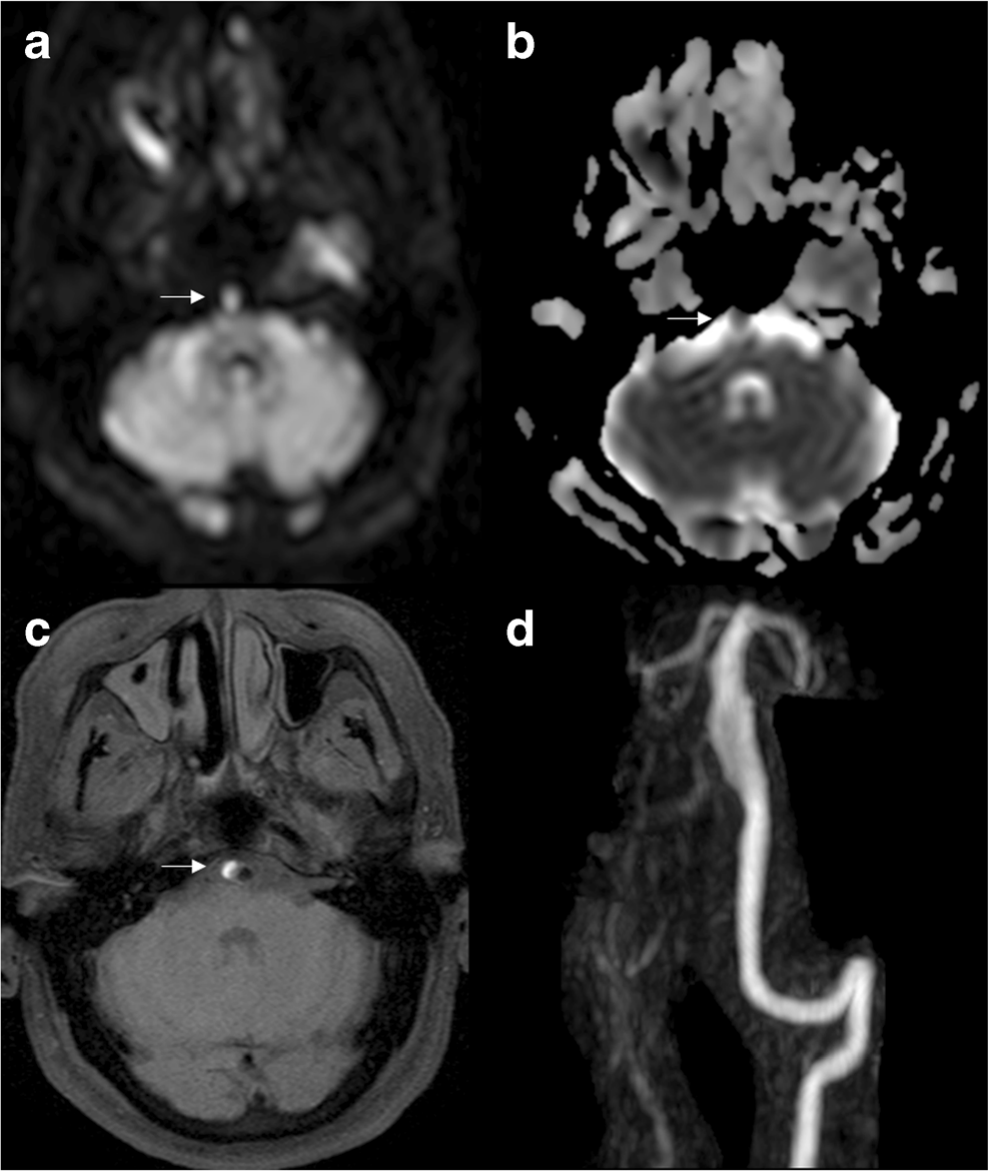
Arterial dissection. DWI (**a**), ADC (**b**), non-contrast FAT SAT T1 (**c**) and coronal MIP reconstruction of the basilar artery (**d**) demonstrate a dissection with mural haematoma represented by the “crescent sign” (arrows), which shows restricted diffusion and hyperintensity on T1WI

The “crescent sign”, representing an intramural haematoma, can be an imaging criterion for diagnosing arterial dissection. It consists of an eccentric rim of hyperintensity surrounding the hypointense arterial lumen on MRI. It has traditionally been described on T1-weighted fat-saturation MRI sequences [26], but may be seen on other sequences such as diffusion-weighted imaging (Fig. 3).

The signal intensity of intramural haematoma in arterial dissection is influenced by the paramagnetic effects of blood breakdown products [27]. The haematoma is isointense on T1WI and shows T2 prolongation a few hours after bleeding has occurred in the hyperacute phase (oxyhaemoglobin phase). The T2 signal becomes hypointense in the acute stage (deoxyhaemoglobin phase). T1 and T2 signals become hyperintense in the subacute phase, between 7 days and 2 months (methaemoglobin phase). In cerebral venous thrombosis, hyperintensity on DWI has been suggested to represent restricted proton movement within the clot, but it is also observed within the dissected vertebral artery wall in the hyperacute and early subacute phases [28]. DWI can therefore be useful in the diagnosis of acute dissection, when the intramural haematoma can barely be detected on fat-saturated T1-weighted images because of obscuration of its isointense signal by the surrounding soft tissues with similar signal intensity.

DWI using EPI usually shows susceptibility artefacts at the air-tissue or bone-soft tissue interfaces. Artefacts present as a fuzzy linear hyperintensity or diffuse hypointensity, which are usually easy to differentiate from the focal round or linear well-demarcated hyperintensity typical of intramural haematoma [28]. Correlation with T1- or T2-weighted images is also helpful. The use of an SE sequence, reducing the echo time and increasing the acquisition matrix, may be helpful to minimise these susceptibility artefacts.

**Table:** 

Key points	DWI helps in the detection of the intramural haematoma
Pearls	Bright spot on the DWI sequence early on (when it might be isointense or harder to see on other sequences)
Pitfalls	Susceptibility artefacts at skull base may interfere with the correct interpretation of this sign on DWI

### Venous infarction

Ischaemia in the setting of venous thrombosis has a different physiology from arterial infarction [29]. Initially, there is increased venous pressure and vasogenic oedema (reversible) that may lead to a Na^+^/K^+^ ATPase pump failure, cytotoxic oedema and restricted diffusion. Therefore, diffusion imaging helps to define the stage of the disease because it allows differentiation between irreversible venous infarction (DWI hyperintense with restricted diffusion) and reversible venous oedema (DWI isointense with increased diffusion). In contradistinction to arterial stroke, DWI-positive venous stroke may be completely reversible.

**Fig. 4:**
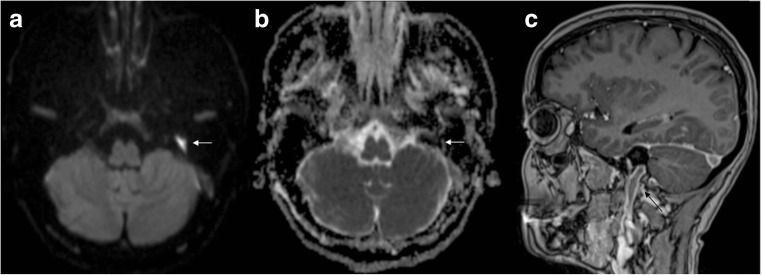
Venous thrombosis. DWI (**a**), ADC map (**b**) and sagittal reconstruction of T1 3D with gadolinium (**c**) show thrombosis of the left jugular vein (arrows), presenting with restricted diffusion and a filling defect in the vessel after contrast administration

In venous ischaemia, DWI may be able to show the actual thrombus as increased signal intensity [30] because of the restricted movement of water molecules within the venous clot (Fig. 4). However, sinus thrombosis may have different appearances depending on the stage of thrombus formation, which has been demonstrated by ex vivo MRI measurements [31].

In previous studies, the approximate frequency of the increased DWI signal within the venous thrombus has been reported as 41% [32], thus not truly providing significant added value since all cases showed T1WI or FLAIR signal changes at the site of occlusion.

Taking into account that venous infarctions have a higher tendency to bleed, and haemorrhage can also restrict diffusion, imaging features in venous infarction may be considerably variable, depending on the time after onset and the sequence of events.

Other vascular pathologies in which diffusion may be helpful are transient global amnesia, with a typical transient focal dot-like area of diffusion restriction in the hippocampus and/or parahippocampal gyrus [33], or posterior reversible encephalopathy syndrome (PRES), where the majority of cases do not show diffusion restriction because oedema is typically vasogenic. In the cases where DWI is positive, this may indicate the earliest sign of non-reversibility due to progression to cytotoxic oedema and therefore the possibility of permanent infarction or haemorrhagic transformation.

**Table:** 

Key points	Different physiology from arterial ischaemia
Pearls	Depending on stage, the thrombus may be hyperintense on DWI
Pitfalls	Very variable imaging features, depending on the time after thrombosis

## Tumours

Regarding extraaxial tumours, DWI allows distinction between epidermoid and arachnoid cysts. Epidermoid cysts show diffusion restriction, as opposed to arachnoid cysts, which follow CSF signal intensity on all sequences. Other MR sequences can also aid in the diagnosis, since epidermoid cysts are bright on FLAIR, but not arachnoid cysts [34]. In meningiomas it has been shown that ADC values correlate with tumour cellularity [35] but this has not been found to provide any additional value in differentiating histopathological subtypes of meningiomas [36].

Concerning glial tumours, quantitative assessment with DWI has mainly targeted an estimation of cellularity based on its inverse relation with water diffusivity in the extracellular compartment. Hence, ADC can be considered as a tumour biomarker in gliomas: the higher the tumour grade, the lower the mean tumour ADC values [37]. However, the range of ADC values within a given glioma varies markedly [38] and there is an overlap between ADCs of grade II astrocytomas and glioblastomas, probably due to the inherent tissue heterogeneity associated with gliomas across different grades, limiting the use of DWI. Despite this, in the context of low-grade tumour follow-up and where appropriate, a decrease in the ADC value could be considered an early sign of tumour progression.

**Fig. 5:**
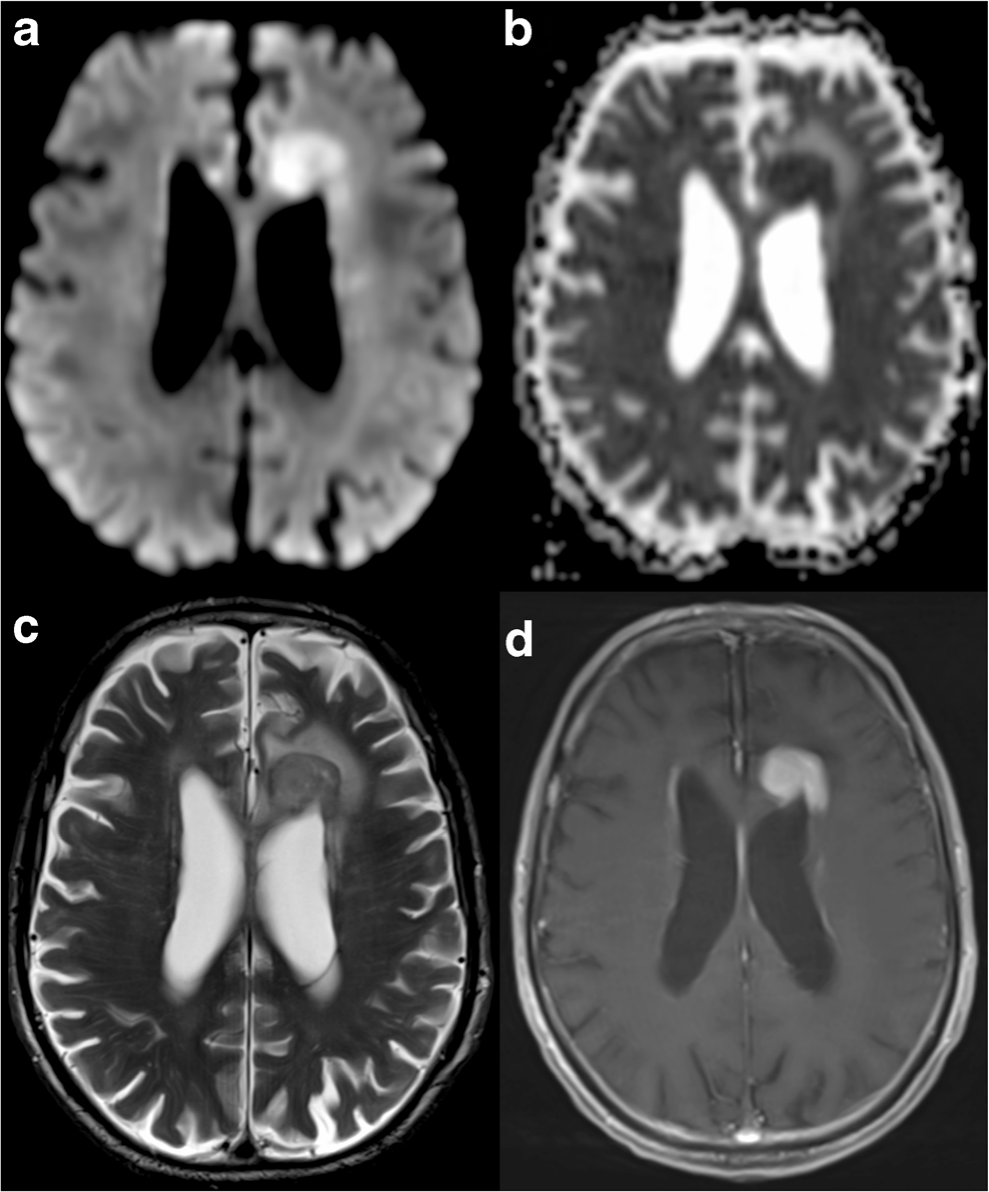
Primary CNS lymphoma. Left frontal periventricular lesion showing prominent diffusion restriction, presenting with hyperintensity on DWI (**a**), low ADC value (**b**) and mild hyperintensity on T2WI (**c**), all typical features of this type of hypercellular tumour. T1WI post gadolinium (**d**) shows homogeneous and intense contrast enhancement

Another use of DWI on imaging of CNS tumours is to aid in the distinction between glioblastoma and primary CNS lymphoma, two entities with markedly different treatment strategies and not always easy to differentiate with conventional imaging in patients presenting with an enhancing brain mass. Due to its higher cellularity compared with GBM, primary CNS lymphoma has been shown to exhibit lower ADC values [39]. There are some published works aiming to find an ADC threshold for differentiating lymphoma from other tumours [40] (Fig. 5). Guzman et al. reported higher ADC values in the more aggressive tumours (comparing glioblastoma and metastasis vs. low-grade glioma) [41].

Attempts to differentiate glioblastoma from metastasis based on the FA values of the surrounding oedema have been made [42] based on the assumption that glioblastoma has a tendency to grow in an infiltrative manner typically invading surrounding tissues. Conversely, metastatic tumours tend to grow in an expansive manner and typically displace the surrounding brain tissues rather than invading them [43]. Another entity associated with brain tumours is intracranial hypertension, which can be seen as papilloedema, bright on DWI [44].

**Fig. 6:**
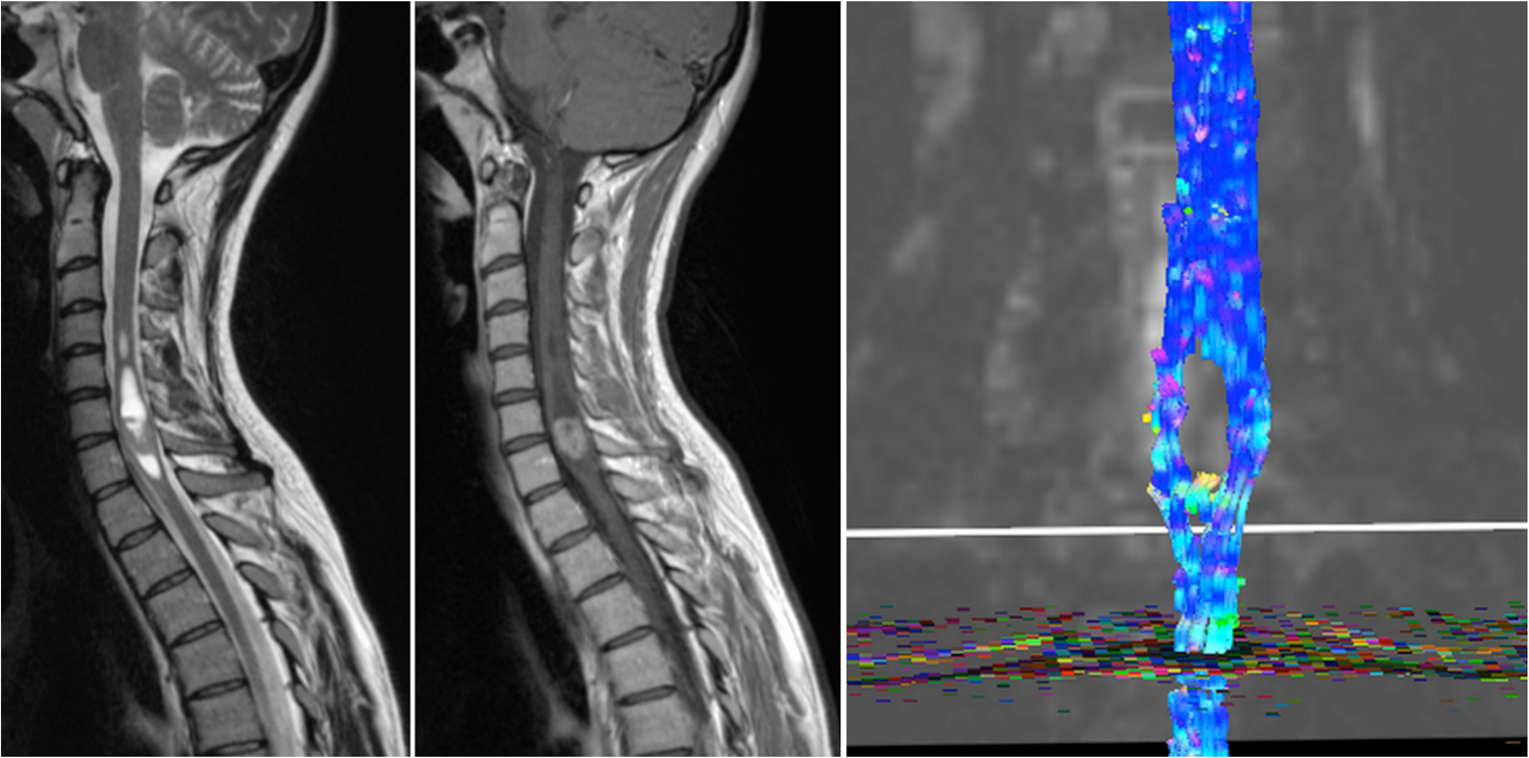
DTI for surgical planning. Patient with an ependymoma in the lower cervical spinal cord with areas of high T2 signal (**a**) and enhancement after gadolinium administration (**b**). DTI shows displacement of the fibres (**c**), which is usually consistent with a slow-growing lesion

DTI and tractography reconstruction can be helpful for surgical planning in patients with brain or spinal cord masses. It shows white matter tracts as well as displacement or interruption of these tracts secondary to different pathologies. Slow-growing tumours displace the fibres surrounding the lesion, whereas in ischaemia or trauma, representing more acute insults, the fibres are usually interrupted [45] (Fig. 6).

**Fig. 7:**
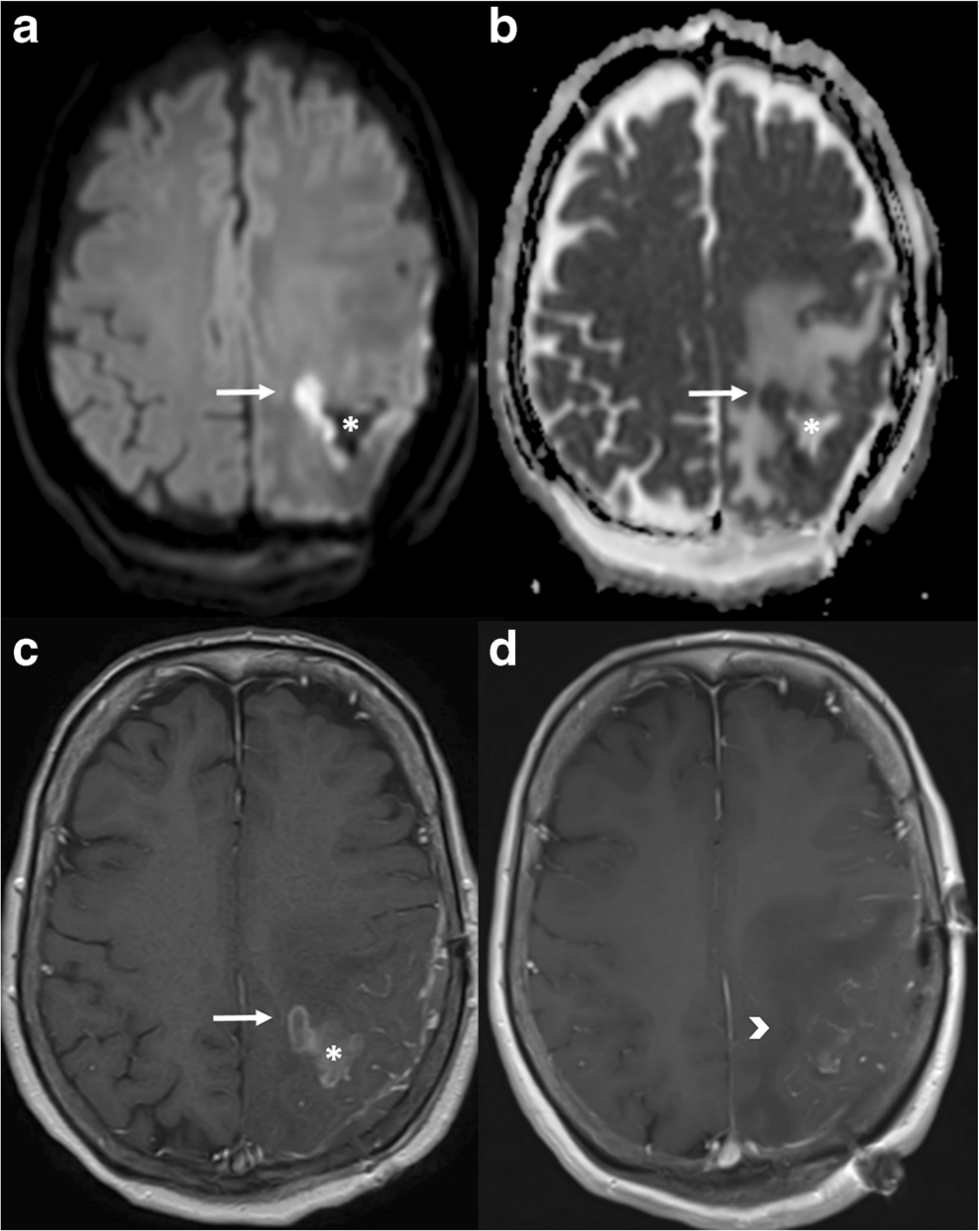
Post-surgical ischaemia. Immediate follow-up MRI in a patient who underwent surgery for resection of a suspicious enhancing mass. In the medial aspect of the resection cavity (asterisk) there is an enhancing area on the T1 post-contrast sequence (**c**, arrow). This finding alone could represent residual tumour, but the presence of restricted diffusion with high signal on DWI (**a**) and a low ADC value (**b**) meant that a small area of peri-surgical ischaemia was more likely. Three-month follow-up T1 post-gadolinium MRI (**d**) shows absence of enhancement in the same region (arrowhead), confirming this diagnosis

An additional important use of DWI after surgical resection of a tumour is to detect areas of diffusion restriction in the cavity margins on MRI performed in the first 48 h, corresponding to small postsurgical infarctions. The importance of this imaging time window lies in the fact that the majority of these infarcts will enhance after 1 or 2 weeks and could be potentially interpreted as residual tumour or tumour progression (Fig. 7).

Regarding paediatric tumours, DWI can non-invasively add valuable information that can be used to narrow the differential diagnosis of the three most common tumours of the posterior fossa in children (medulloblastoma, ependymoma, pilocytic astrocytoma). Several studies have demonstrated that ADC values in the enhancing, non-necrotic, non-oedematous, solid parts of cerebellar tumours are negatively correlated with tumour grade [46] (high-grade tumours such as medulloblastoma are characterised by high cellularity, low extracellular space, and cells with large nuclei and high nuclear-to-cytoplasmatic ratios, causing decreased diffusion). Cutoff values of > 1.4 × 10^3^ mm^2^/s for juvenile pilocytic astrocytoma and < 0.9 × 10^3^ mm^2^/s for medulloblastoma have been suggested with a high specificity [47]. An even greater accuracy can be achieved by combining DWI and MRS to obtain more information about the tumour proliferative potential [48]. DWI has also been reported to be useful in detecting relapse of embryonal tumours such as medulloblastoma, being more sensitive than contrast-enhanced MRI in subjects with the classic variant [49].

**Table:** 

Key points	ADC is inversely proportional to tumour cellularity
Pearls	DWI can help differentiate GBM from lymphoma, epidermoid from arachnoid cysts, tumour necrosis from tumour progression and in narrowing the differential diagnosis of posterior fossa tumours in children, to name a few
Pitfalls	Overlaps and exceptions. Always interpret in the light of the clinical context and other radiological information

## Infection and inflammatory conditions

### Infection

**Fig. 8:**
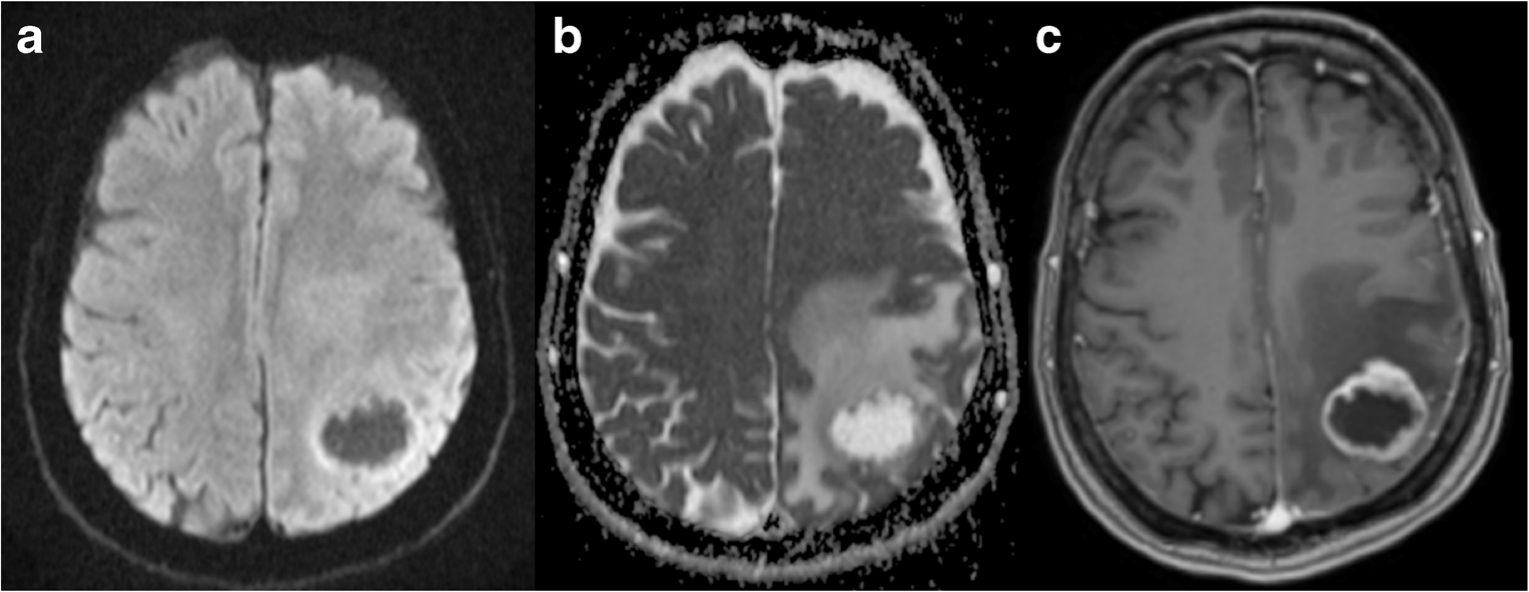
GBM. Left parieto-occipital lesion with peripheral vasogenic oedema. DWI and ADC (**a** and **b**, respectively) show a clear area of increased diffusion within the core, corresponding to a necrotic centre. On T1 3D post gadolinium the mass shows ring enhancement

**Fig. 9:**
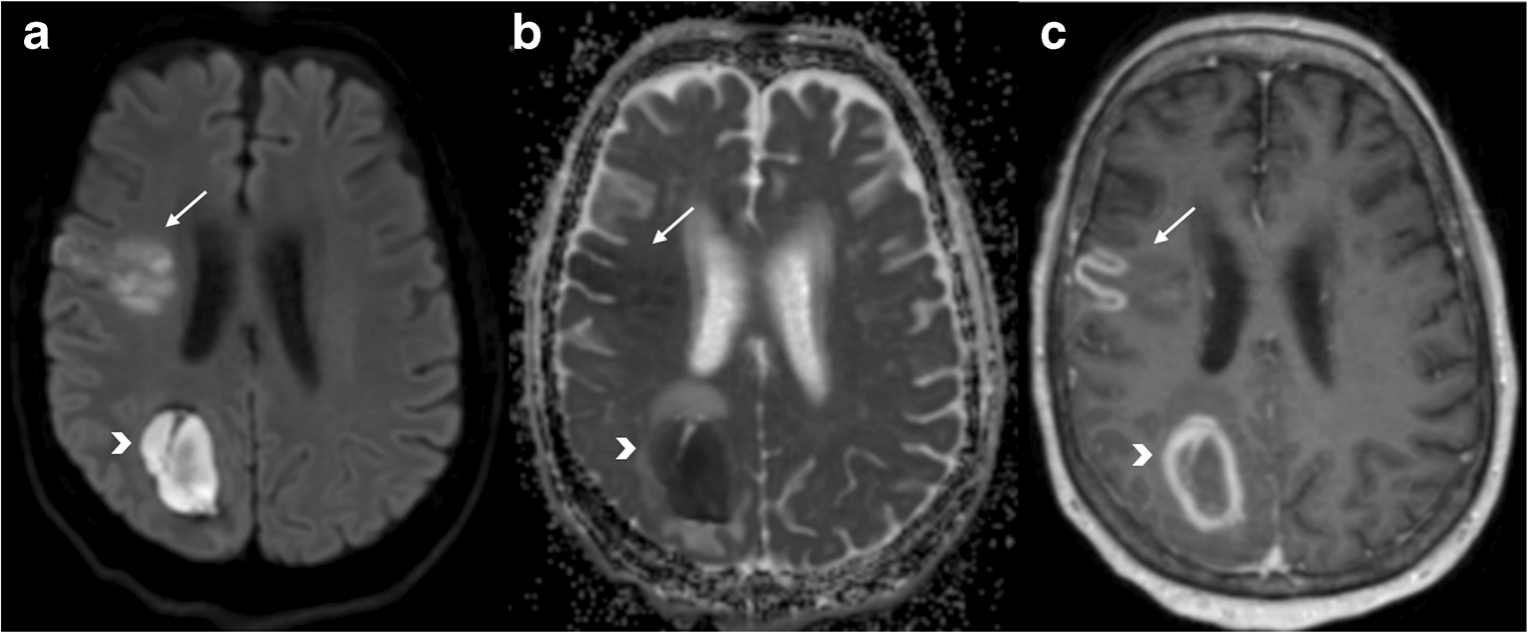
Subacute haematoma. Right parietal mass (arrowhead) showing diffusion restriction within the core on DWI and ADC maps (**a** and **b**, respectively) and a ring-enhancing pattern on T1 post gadolinium (**c**). This was a subacute haematoma. Clinical context is important to differentiate haemorrhage from abscess. There is also a subacute ischaemic lesion in the inferior right frontal lobe (arrows) that shows early pseudonormalisation of the ADC and gyriform enhancement post gadolinium

Abscesses of the brain can be bacterial, parasitic or fungal in origin. The appearance of bacterial abscesses on conventional imaging varies with the stage of abscess formation, but the typical features of the early capsule stage (ring-enhancing lesion with a T2 hypointense rim and variable degree of associated vasogenic oedema) can be similar to primary cystic or necrotic tumours (Fig. 8) and subacute haematomas (Fig. 9). However, pyogenic abscesses show restricted diffusion with markedly decreased signal on the ADC map and elevated fractional anisotropy (FA) within the abscess cavity [50] because of the presence of intact inflammatory cells and bacteria. These impede the microscopic motion of water molecules, which helps to narrow the differential diagnosis of ring-enhancing lesions (exceptions exist: metastases with high cellularity and haemorrhagic metastasis).

**Fig. 10:**
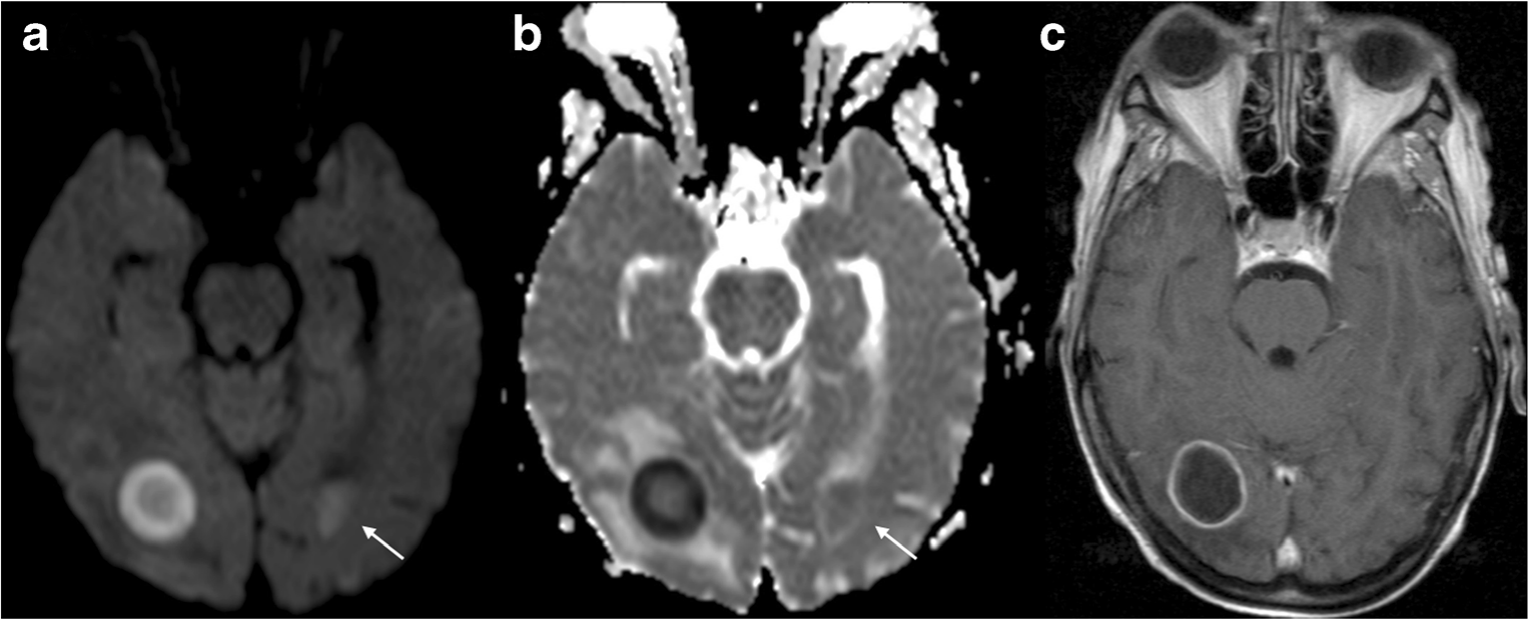
Abscess. Right occipital mass showing marked diffusion restriction within the core on DWI and ADC maps (**a** and **b**, respectively) and a peripheral enhancing pattern on T1 post gadolinium (**c**). DWI helps to differentiate ring-enhancing lesions because restricted diffusion in the centre of the mass is characteristic of pyogenic abscesses. In this case, diffusion-based sequences also helped to identify ventriculitis (arrows)

Reddi et al. showed that diffusion-weighted imaging had a sensitivity and specificity of 96% for the differentiation of brain abscesses from primary or metastatic cancers (positive predictive value, 98%; negative predictive value, 92%) [51] (Fig. 10). A study [52] has shown that the abscess cavity had lower ADC and higher FA compared with the cystic cavity of glioblastomas and metastases, possibly representing high viscosity and organised viable inflammatory cells. In addition, FA values were higher in the enhancing rim of the abscess than in glioblastoma and metastasis, maybe because of the presence of concentric layers of collagen fibres. Regarding the peritumoral zone of oedema, this study showed that the presence of a hyperintense FA rim (with lower FA values) was much more frequent in glioblastomas and metastases than in abscesses. This suggests that microstructural changes in the oedematous white matter immediately surrounding the abscess may be different from those surrounding glioblastoma and metastasis, possibly because of acute versus longer time to formation, respectively.

Fungal abscesses can have either restricted or elevated diffusion in the centre (depending on the content), in opposition to pyogenic and tuberculous abscesses that typically show restricted diffusion in the core of the cavity [53]. Restricted diffusion is usually demonstrated in the projections and wall of the fungal abscess, referred to as “intracavitary projections”, and represent the fungal organism itself.

Aspergillus CNS infections tend to be haemorrhagic, which is an additional cause of restricted diffusion. In general, it is important to include fungal abscesses in the differential diagnosis in immunocompromised patients because of different targeted antifungal treatment.

Parasitic abscesses may also show both restricted and elevated diffusion [54]. For example, cysticercosis cysts have a similar or slightly increased DWI signal to CSF, and the scolex may be seen as a hyperintense focus inside [55]. In toxoplasmosis, diffusion restriction is highly variable (possibly overlapping with lymphoma characteristics, its main differential diagnosis) [56, 57]. In cerebral malaria, DWI is useful in detecting areas of brain infarction [58].

In the postsurgical setting, the accuracy of DWI in the diagnosis of CNS infectious complications is low, showing a high false-negative rate. There are often postoperative changes with diffusion restriction due to haemorrhage/ischaemia, which can hinder the distinction between purulent content and haemorrhagic/ischaemic components. The absence of restricted diffusion is not sufficient to exclude the presence of pyogenic postcraniotomy infection and should not be used as the main determinant in patient management in this clinical setting [59].

Diffusion restriction is commonly seen in viral encephalitis, and the location of the affected area may guide the radiologist in the identification of the infectious agent. For example, the limbic system (medial temporal and inferior frontal cortex) is the typical involved area in herpes simplex encephalitis. Viruses more frequently causing potentially reversible lesions in the splenium of the corpus callosum are the influenza virus [60], Epstein-Barr virus, HHV-6 and papovirus JC [61]. Thalamic involvement is typically seen with the West Nile virus [62], Japanese encephalitis or Eastern equine encephalitis. DWI may help detect lesions earlier than conventional MR imaging. In patients diagnosed with West Nile virus encephalitis, diffusion restriction with no FLAIR or T2 signal abnormalities has been reported to be a sign of good prognosis [63].

Progressive multifocal leukoencephalopathy (PML) is secondary to the infection of oligodendrocytes by the JC polyoma virus, which typically remains latent until reactivation in the context of an immunocompromised state (HIV, natalizumab or other immunosuppressive treatments). Due to its ability to investigate white matter architecture and diseases, DWI shows regions of active infection and cell swelling with high signal and allows distinction of two parts in the lesions: a central core (whose size can correlate to the clinical status and disease duration) and a peripheral rim (which includes a heterogeneous component and areas of surrounding cytotoxic oedema with low ADC and areas of vasogenic oedema and glial repair with intermediate ADC) [64].

**Table:** 

Key points	Inflammatory cells and bacteria within an abscess core restrict diffusion
Pearls	ADC values and morphology within the area showing restricted diffusion may help in differentiating abscess from tumour and pyogenic from fungal abscess The location of diffusion restriction may orientate in the aetiology of viral encephalitis: limbic system for herpes simplex; splenium of the corpus callosum for influenza and Epstein-Barr viruses; thalami for West Nile virus DWI most sensitive technique in the early stages of CJD
Pitfalls	Subacute haematoma can look like an abscess

### Creutzfeldt-Jakob disease

Creutzfeldt-Jakob disease (CJD) is the combination of progressive dementia and pyramidal, extrapyramidal, and/or cerebellar signs caused by a prion (proteinaceous particle without DNA or RNA but capable of causing infection). DWI MR has high diagnostic accuracy and is the most sensitive neuroimaging technique for striatal and cortical lesions from an early stage [65]. Typically, diffusion-weighted hyperintensity is progressive and persistent over many weeks, affecting the striatum and cortex. The gyriform DWI-hyperintense areas in the cerebral cortex (“cortical ribbon” sign) correlate to the location of periodic sharp-wave complexes on EEG [66]. DWI hyperintensity may resolve late in the disease.

The pulvinar sign (symmetrical hyperintensity in the posterior thalamic nuclei on DWI or T2WI), “hockey stick” sign (signal changes affecting the dorsomedial thalamic nuclei) or similar changes in the periaqueductal grey matter were reported to be specific for variant (vCJD) [67], but can also occur, although less frequently, in sporadic (sCJD).

### Multiple sclerosis

In multiple sclerosis, the majority of active plaques show normal or increased diffusivity, but some may show restricted diffusion, often at the margins [68]. It is important to bear this in mind since, in the appropriate context, these plaques can mimic a lacunar infarct. Possible explanations for the decreased ADC in active plaques are decreased extracellular space due to myelin oedema or cytotoxic oedema and decreased water movement in the extracellular space secondary to inflammation [15]. DTI demonstrates different degrees of mean diffusivity increase and FA decrease in T2-hyperintense established lesions or even before their formation. These changes are also present in normal-appearing white or grey matter of patients with MS [69] (as opposed to ADEM, which shows normal diffusivity within normal-appearing white matter). Diffusivity changes in MS correlate with areas of demyelination and axonal loss in postmortem studies [70].

### Immune-mediated encephalitis

Immune-mediated encephalitis includes two different types of conditions: paraneoplastic encephalitis syndromes, comprising limbic encephalitis, paraneoplastic cerebellar degeneration and brainstem encephalitis, and the encephalitis syndromes associated with antibodies against neuronal cell surface/synaptic proteins, which are commonly referred to as autoimmune encephalitis.

Diagnosis is based on electroencephalography (EEG), lumbar puncture and serological testing for appropriate biomarkers (presence of autoantibodies, lymphocytic pleocytosis or oligoclonal bands in CSF). Neuroimaging should be performed despite yielding a high proportion of false negatives (in autoimmune encephalitis more than in paraneoplastic encephalitis syndromes) [71]. When positive, the studies are usually non-specific (high FLAIR signal in cortical or subcortical regions—hippocampus, basal ganglia, white matter). Diffusion restriction may be present in the areas showing abnormal signal.

Although limbic encephalitis has been known as a paraneoplastic syndrome, there have been recognised forms characterised by serum autoantibodies, directed against glutamic acid decarboxylase (anti-GAD) and voltage-gated potassium channels (VGKC), not related to neoplastic conditions.

On DTI studies, a reduction of fractional anisotropy and increased mean diffusivity have been shown in the white matter of patients suffering from NMDAR encephalitis (the most common type of autoimmune encephalitis), suggesting that white matter damage is a contributor to the pathophysiology and clinical phenotype, even correlating with disease severity [72].

Another study aiming to assess white matter integrity in the two forms of non-paraneoplastic limbic encephalitis characterised by serum autoantibodies (GAD and VGKC) concluded that GAD-limbic encephalitis presented with FA reduction, probably reflecting demyelination, whereas VGKC-limbic encephalitis caused no changes in the diffusion properties of white matter, probably because this condition is more limited to grey matter [73].

### Neuritis

**Fig. 11:**
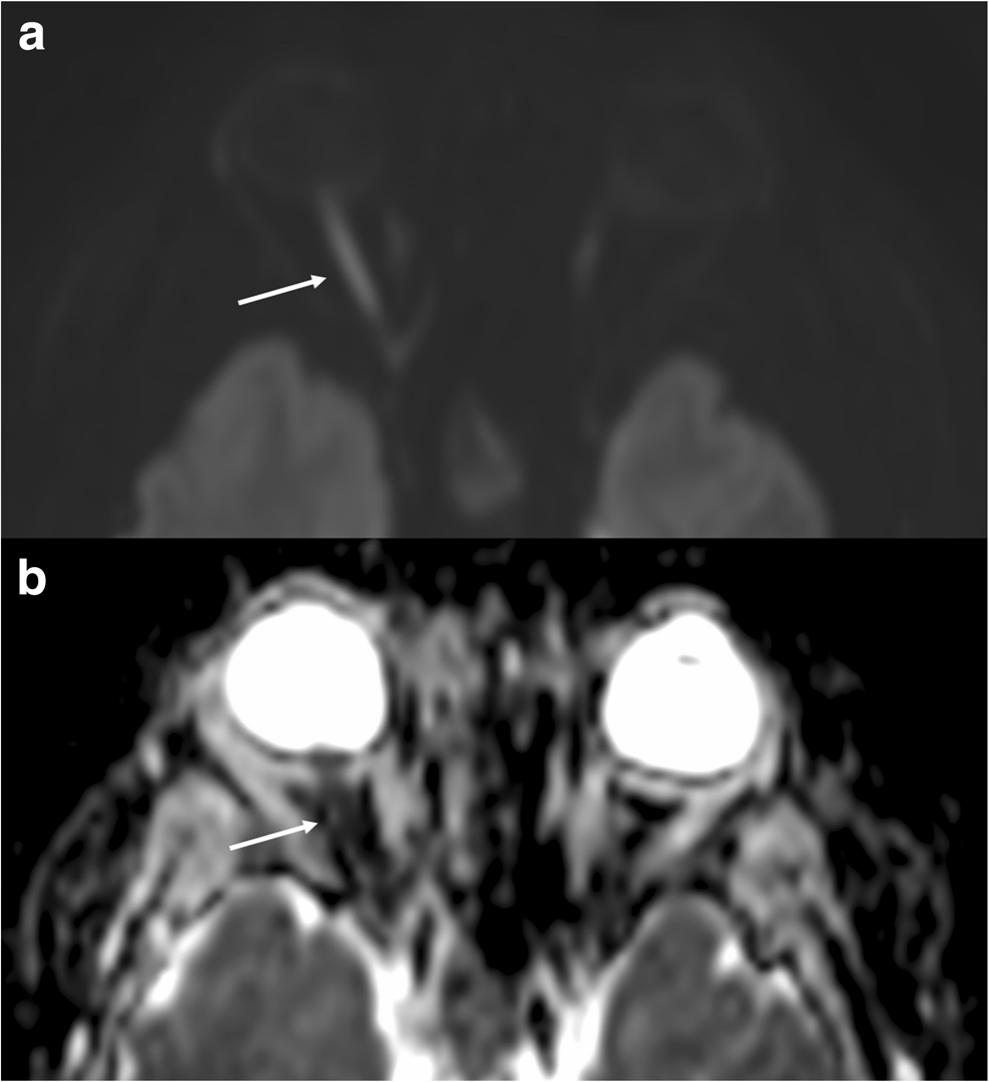
Optic neuritis. High DWI signal (**a**) and low ADC value (**b**) representing restricted diffusion in the right optic nerve in a patient with non-specific optic neuritis

Abnormal signal intensity of the optic nerve due to diffusion restriction may be seen in ischaemic or traumatic optic neuropathy [74, 75]. DWI has been suggested as a useful tool in differentiating these aetiologies from acute optic neuritis (presumed autoimmune aetiology) [76, 77], where there is increased diffusivity in demyelinating plaques, related to disruption of myelinated axons, and decreased fractional anisotropy. However, this should be interpreted in the clinical context, since optic neuritis may also show transient decreased diffusion in the acute phase (Fig. 11).

With the exception of the orbital apex region, DTI of the optic nerve is facilitated by its anatomy and antero-posterior orientation. This can be useful in assessing tumours such as neurofibromas, schwannomas or meningiomas [78] as well as in brain malformations such as septo-optic dysplasia or optic nerve hypoplasia [79].

Other cranial nerves can also be studied with high-resolution DTI in some clinical situations. Demonstration of atrophy or loss of anisotropy in cases of trigeminal neuralgia and fibre displacement in vestibulocochlear schwannomas are examples of this [80].

## Neurodegenerative disorders

The DWI and DTI techniques allow quantitative analysis of microstructural changes in neurodegenerative diseases, even when no abnormalities are seen on conventional MRI sequences, because they are able to identify changes in brain tissue integrity. These changes are characterised by a decrease in the number of barriers that restrict the movement of water, thus causing ADC to increase in brain areas where neurodegeneration occurs. This has led to an increase in the use of DWI in the diagnostic investigation of neurodegenerative parkinsonian syndromes. Attempts have been made to use DTI of the substantia nigra as a diagnostic marker for Parkinson disease, but while some studies found highly significant FA reduction in the substantia nigra [81], others concluded that there is not sufficient evidence for nigral DTI parameters to serve as biomarkers for Parkinson’s disease [82].

ADC values of the putamen have been used to discriminate between Parkinson’s disease and atypical parkinsonism, including the classical phenotype of progressive supranuclear palsy and corticobasal syndrome. Putaminal ADC values were significantly higher in both atypical parkinsonism syndromes compared with Parkinson’s disease [83].

In other neurodegenerative diseases, such as Friedreich’s ataxia, DWI may be an appropriate non-invasive tool to systematically quantify the extent and distribution of brain changes. DWI has also highlighted the involvement of new areas in the pathophysiology of this condition, such as the optic radiations and middle cerebellar peduncles [84].

## Conclusion

Diffusion-weighted sequences currently play a central role in neuroimaging. The already widespread qualitative assessment allowed by these sequences improves sensitivity in the depiction of several central nervous system conditions, whereas new models of diffusion sequences provide quantitative parameters, allowing potential biomarkers for diagnosis, prognosis and follow-up. The future of DWI will undoubtedly include technical improvements to enhance data fidelity, to achieve high isotropic resolution (e.g., submillimetre) for 3D acquisitions and to reduce the image acquisition time. Furthermore, advances in ultrahigh field technology are already being applied to DWI.
